# UHPLC-Q-TOF/MS-Based Metabolomics Approach Reveals the Antifungal Potential of Pinocembroside against *Citrus* Green Mold Phytopathogen

**DOI:** 10.3390/plants9010017

**Published:** 2019-12-22

**Authors:** Chuying Chen, Nan Cai, Jinyin Chen, Chunpeng Wan

**Affiliations:** 1 Jiangxi Key Laboratory for Postharvest Technology and Nondestructive Testing of Fruits & Vegetables, Collaborative Innovation Center of Postharvest Key Technology and Quality Safety of Fruits & Vegetables in Jiangxi Province, Jiangxi Agricultural University, Nanchang 330045, China; cy.chen@jxau.edu.cn (C.C.); wq1252733770@163.com (N.C.); 2 Pingxiang University, Pingxiang 337055, China

**Keywords:** *Penicillium digitatum*, pinocembroside, green mold, antifungal mechanism, membrane peroxidation

## Abstract

Pinocembroside (PiCB) isolated from *Ficus hirta* Vahl. fruit was studied herein with the aim to find the potential mechanism for significant inhibition of growth of *Penicillium digitatum*, a causative pathogen of citrus green mold disease. PiCB substantially inhibited mycelial growth of *P. digitatum*, with the observed half maximal effective concentration (EC_50_), minimum inhibitory concentration (MIC), and minimum fungicidal concentration (MFC) of 120.3, 200, and 400 mg/L, respectively. Moreover, PiCB altered hyphal morphology and cellular morphology by breaking and shrinking of mycelia, decomposing cell walls, cytoplasmic inclusions. In addition to, a non-targeted metabolomics analysis by UHPLC-Q-TOF/MS was also performed, which revealed that PiCB treatment notably disrupted the metabolisms of amino acids, lipids, fatty acids, TCA, and ribonucleic acids, thereby contributing to membrane peroxidation. Current findings provide a new perception into the antifungal mechanism of PiCB treatment in inhibiting *P. digitatum* growth through membrane peroxidation.

## 1. Introduction

Citrus fruit is a major economic crop with a diverse range of species and varieties, including oranges, mandarins, tangerines, pummelos, lemons, citrons, kumquats, limes, and different hybrids [1,2]. Postharvest storage and shelf life of citrus fruit are limited due to the attack of several pathogens such as *Penicillium* spp., *Geotrichum citri-aurantii*, *Alternaria alternata*, and *Diaporthe citri* being the prevalent postharvest pathogenic fungi in citrus fruit [3,4,5,6]. Among these pathogens, *P. digitatum*, the causal agent of green mold, is considered one of the most threatening postharvest diseases in citrus fruit. The *P. digitatum* infection on citrus fruit has posed a series of adverse effects including toxins accumulation, postharvest deterioration, human health, environmental pollution, and 60–80% of postharvest crop loss in China [7,8]. To reduce the incidence of green mold, a variety of synthetic fungicides have been commonly used to ensure a stable supply of citrus fruit at markets for a growing world population [9,10]. However, the use of synthetic fungicides has received worldwide concern due to their potential undesirable biological effects in humans, animals, and the environment. Moreover, such synthetic antifungal agents remain as toxic residues and can cause public health concerns, the emergence of resistant fungi, and environmental pollution [11,12]. Keeping in view these issues, the control of citrus green mold using various natural antifungal agents sourced from medicinal plants are thought to be the superior choice than synthetic agents in order to prevent postharvest green mold disease in citrus fruit.

**Figure 1:**
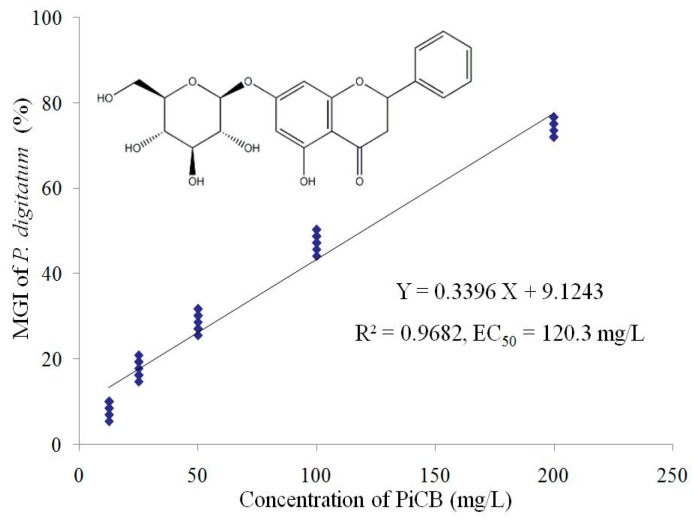
Linear regression analysis between pinocembroside (PiCB) concentration and mycelial growth inhibition (MGI) of *Penicillium digitatum*. The linear regression model (*Y* = 0.3396*X* + 9.1243) and coefficient of determination (*R*^2^ = 0.9682) are shown. The chemical structure of PiCB as an inset is embedded in Figure 1.

During the last decades, the use of natural antifungal components has been a novel alternative for the traditional synthetic fungicides used to reduce postharvest fungal rotting of citrus and other fresh fruits. In keeping with this global trend, our lab has been exploring novel antifungal phytochemicals that help reduce disease incidence and prolong the storage life of citrus fruit [13]. Among various natural phytochemicals, pinocembroside (5,7-dihydroxyflavanone-7-O-β- D-glucoside, PiCB) (Figure 1) is a natural flavonone reported to be found in *F. hirta* Vahl., *Penthorum chinense* Pursh, and *Viscum articulatum* [14,15,16]. PiCB, a flavonoid glycoside, was isolated as the major antifungal compound in *F. hirta* Vahl. fruits, which is a widely used traditional medicine and a popular ingredient in soups in southern China [14,17,18]. Recently, PiCB has drawn attention due to its strong preventive effects against hepatic steatosis and significant protection against chronic ethanol-induced liver injury [19]. PiCB showed antifungal potential against *P. italicum* in PDA media, which depicted that the drug successfully inhibits the mycelial growth of *P. italicum* [14].

However, there is hardly any authentic and detailed study regarding the antifungal activity of PiCB against *P. digitatum* in citrus fruit. In order to depict the changes in hyphal morphology and metabolism level in *P. digitatum* treated with PiCB, we used microscopy and UHPLC-Q-TOF/MS-based non-targeted metabolomics. The current study aimed to find the differential metabolites of *P. digitatum* response to PiCB, extending to explore the potential mechanism of PiCB on antifungal effects of *P. digitatum*.

## 2. Results and Discussion

### 2.1. Effect of PiCB on Mycelial Growth of P. digitatum

The inhibitory effects of PiCB on mycelial growth of *P. digitatum* showed interesting results (Figure 1). The mycelial growth of *P. digitatum* on PDA medium was significantly inhibited by PiCB in a dose-dependent manner—the higher PiCB concentration, the higher MGI. PiCB inhibited over one-quarter of the *P. digitatum* mycelial growth at comparatively low concentration of 50 mg/L, while 200 mg/L inhibited approximately three-quarters (74.03% MGI) of the *P. digitatum* mycelial growth. Furthermore, by calculating the MGI under different PiCB concentrations, a linear regression of the MGI of *P. digitatum* (*Y*) on the log-transformed PiCB concentrations (*X*) was determined (*Y* = 0.3396*X* + 9.1243, *r* = 0.9682) (Figure 1). According to this toxic regression equation, the half maximal effective concentration (EC_50_) of PiCB against *P. digitatum* was 120.3 mg/L, which indicated the mycelial growth of *P. digitatum* was inhibited effectively at low PiCB concentration. In the current study, the MGI of *P. digitatum* was obviously improved by increasing the PiCB concentrations and incubation days (Figure 1). Similar results of various other botanical fungicides such as antofine, carvacrol, citral, limonene, and tannic acid reported a successful control of green mold in postharvest citrus fruit caused by *P. digitatum* [4,7,20,21,22].

**Figure 2:**
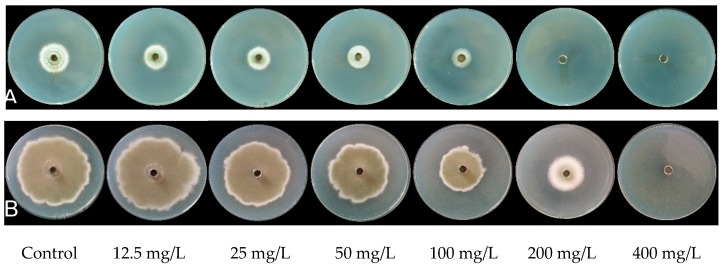
The minimum inhibitory concentration (MIC) and minimum fungicidal concentration (MFC) of PiCB against *P. digitatum* at second day (**A**) and sixth day (**B**) of inoculation.

The value of MIC and MFC is used as an important indicator for assessing the inhibitory efficacy of antifungal agents. Based on the observation made on the *P. digitatum* mycelial growth on PDA medium incubated at 25 °C having various PiCB concentrations (0, 12.5, 25, 50, 100, 200, and 400 mg/L), 200 and 400 mg/L treatments completely inhibited *P. digitatum* mycelial growth on second and sixth day of incubation, respectively (Figure 2). Concurrently, the image of Figure 2 intuitively showed that the higher concentration of PiCB had higher MGI of *P. digitatum*. The MIC and MFC values of PiCB against *P. digitatum* were observed best at 200, and 400 mg/L, respectively, and interestingly much lower than that reported for citronellal, harmol and limonene [20,23,24]. This indicated that PiCB displayed an excellent antifungal efficacy against *P. digitatum*.

### 2.2. Effects of PiCB on Mycelial Weights and Water-Retention Rate of P. digitatum

The effects of PiCB on mycelial weights and water-retention rate of *P. digitatum* are much astonishing and tabulated (Table 1). Higher concentration of PiCB resulted in decreased mycelial weights as well as water-retention rate (Table 1). Moreover, a marked decline (*p <* 0.05) in the mycelial weights (wet weight as well dry weight) of *P. digitatum* followed treatment with a lower concentration (12.5 mg/L) of PiCB.

**Figure 3:**
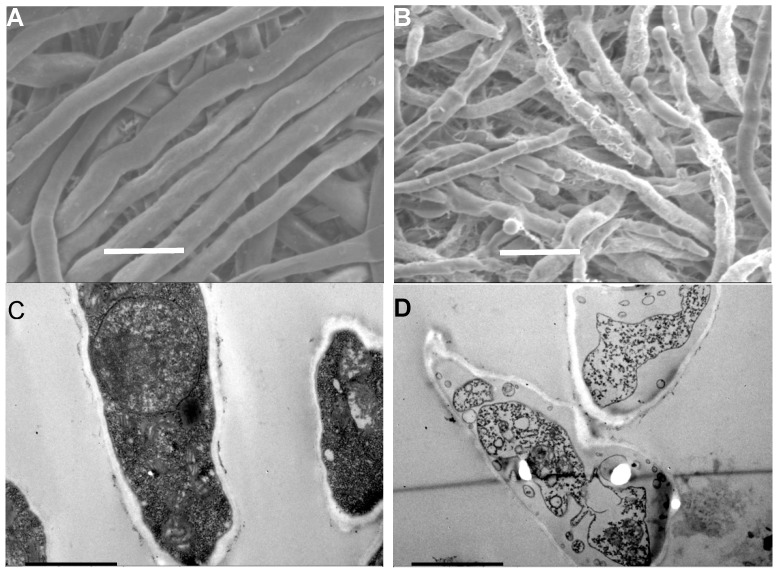
Effects of PiCB on hypha morphology and cell ultrastructure. (**A**) *P. digitatum* mycelia (Control); (**B**) *P. digitatum* mycelia treated with PiCB (100 mg/L). Images observed using scanning electron microscope (SEM) (2500×). Bar scale = 10 μm. (**C**) *P. digitatum* cells (Control); (**D**) *P. digitatum* cells treated with PiCB (100 mg/L). Images observed using TEM (20,000×). Bar scale = 1 μm.

The water-retention rate is used as an important index to evaluate the actual phase of mycelial growth (Table 1). It is clear that increasing concentration of PiCB can significantly decrease water-retention rate of *P. digitatum* (*p* < 0.05). Moreover, the water-retention rate decreased with increasing PiCB concentration from 25 to 400 mg/L, but 200 mg/L concentration keep 83.4% water-retention rate and is close enough to the 400 mg/L PiCB-treated group. This data is in conformity with our previous reports where carvacrol showed positive effects in the MGI of *P. digitatum* [4]. The amount of water and its vital movements inside tissues have a close relationship; thus, rates of water-retention described the cell membrane permeability [25,26]. In the present study, the cell membrane damage in *P. digitatum* caused by PiCB, as evidenced by a decrease in water-retention rate, was accompanied by a marked breakage and leakage of the cell membranes (Table 1 and Figure 3). Current findings mimicked similar studies involving natural antifungal agents for various fungal strains, but the present results showed that the water electrolyte imbalance is sufficient to induce the leakage of intracellular electrolytes leading to cell death [27,28].

### 2.3. Effect of PiCB on Hyphal Morphology of P. digitatum Mycelia

To explore the antifungal mechanism of PiCB against *P. digitatum*, the hyphal morphology and ultrastructure of *P. digitatum* mycelia and cells treated with PiCB were observed by scanning electron microscope (SEM) and transmission electron microscope (TEM), respectively. The SEM images revealed that the hyphal morphology of the control mycelia was normal with a uniform thickness and smooth surface (Figure 3A), while the mycelia treated with PiCB at 1/2 MIC (100 mg/L) caused the formation of a plicated surface and irregular thickness, which were accompanied by production of abnormal offshoots at the top (Figure 3B).

The antifungal mechanism by which PiCB inhibits the mycelial growth of *P. digitatum* is very complicated. In the current study, the SEM images (Figure 3A,B) show the obvious morphological difference between the PiCB-treated and untreated *P. digitatum* mycelia. The results showed that a plicated surface, inflated tip, and sunken mycelia of hyphae emerged in PiCB-treated group. Similar studies demonstrated that the morphological alterations in the plasma membrane are possibly due to the increase in cell membrane permeability, the leakage of small molecular substances, and discrepancies in cell metabolism, resulting in the impairment of the cell membrane [26,29]. Furthermore, the TEM images provided insights about the intracellular structures in PiCB-treated groups of *P. digitatum* cells.

### 2.4. Effect of PiCB on Ultrastructure of P. digitatum Cells

TEM images were also acquired to reveal the antifungal mechanism of PiCB as a superior antifungal agent than the traditional synthetic or already known botanic antifungal agents (Figure 3). The *P. digitatum* mycelia without exposure to PiCB grown in PDB presented a typical fungal cell structure with an abundant cytoplasmic matrix and ordered division (Figure 3C). By contrast, these normal cell structures were conspicuously altered when *P. digitatum* was exposed to various concentration of PiCB. The mycelia treated with PiCB at 1/2 MIC (100 mg/L) exhibited notable shrinking, thinned cell walls, an insufficient cytoplasmic matrix, and reduced cell permeability (Figure 3D), reflecting the leakage or rupture of cell walls to disrupt cytoplasm, thus resulting in cell death.

TEM images clearly differentiate between the PiCB-treated and untreated *P. digitatum* cells (Figure 3C, D). The normal cell structures of untreated *P. digitatum* cells were irreversibly disrupted after the PiCB treatment, which is in line with previous reports describing how the antifungal mechanisms of natural compounds could easily leak out the cytoplasmic matrix (water, protein, sugar, salts, organelles, and other components) and induce aggravation of cell permeabilization [30,31,32].

### 2.5. Effects of PiCB on the Metabolic Profiles of P. digitatum Mycelia

To understand the changes in level of metabolites in *P. digitatum* mycelia exposed to PiCB at 1/2 MIC (100 mg/L), A volcano plot of all metabolite ions is shown in Figure S1. A total of 85 differential metabolites were identified by UHPLC-TOF-MS in *P. digitatum* mycelia. These metabolites were significantly changed in the relative abundance in the PiCB-treated mycelia compared with the control group. Among these metabolites, 23 amino acids, 13 lipids, 10 fatty acids, 3 TCA metabolites, 4 glycolysis/gluconeogenesis metabolites, 3 galactose metabolites, 6 carbohydrate metabolites, 4 nucleic acids, 3 amino sugars and nucleotide sugars, 6 purines, 3 vitamins, and 7 miscellaneous metabolites were observed. Among these differential metabolites, 56 metabolites were up-regulated while 29 metabolites were down-regulated. Interestingly, 18 differential metabolites (α-linolenic acid, uracil, hypoxanthine, 2’-O-methyluridine, LPE 16:0, xanthine, nicotinate, L-phenylalanine, L-methionine, L-tryptophan, sucrose, DGL, L-glutamate, L-aspartate, L-histidine, D-maltose, maltotriose, and L-arginine) were observed in both positive and negative modes (Table S1).

**Figure 4:**
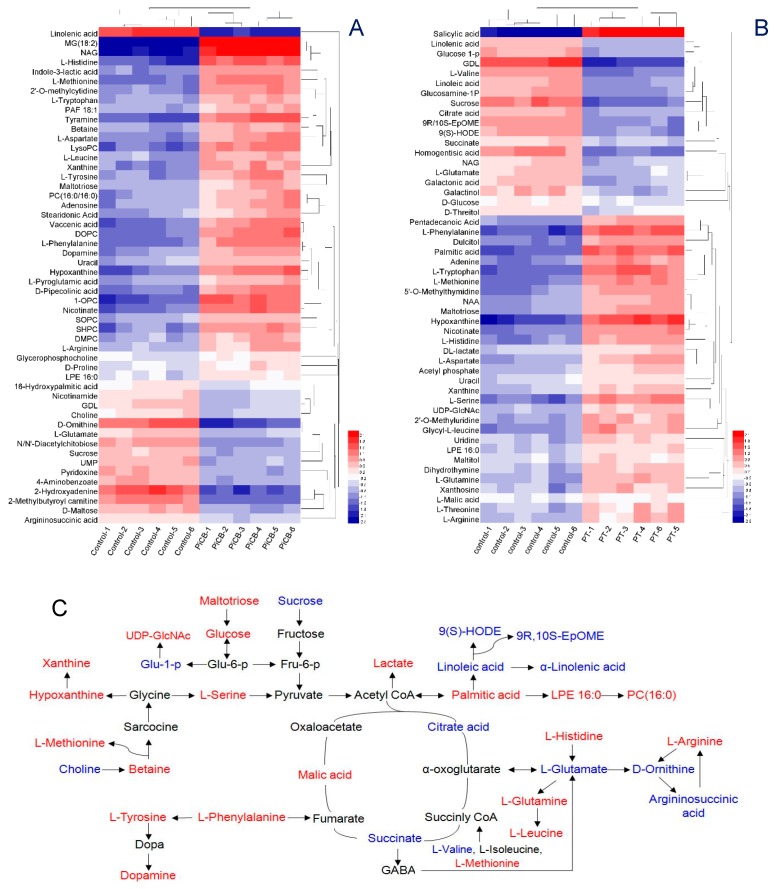
Heat maps of metabolites level change in the PiCB-treated and control groups. Metabolites detected by positive mode (**A**), metabolites detected by negative mode (**B**), proposed metabolism pathways of PiCB against *P. digitatum* (**C**). The data were converted to log base 2 ratios and then subjected to HCA with euclidean distance measures. Each rectangle in heatmap represents a metabolite and its content is colored based on a normalized scale from −2.5 (low, blue color) to 2.5 (high, red color). Red represents increased and blue represents decreased concentrations of the metabolites (*p* < 0.05).

The metabolite profiling of the PiCB treated groups was also investigated with the respective controls. The PiCB-treated and controls were made with six repeats to draw a hierarchical cluster analysis (HCA) (Figure 4). In the positive mode, 52 metabolites were divided into two groups. Up-regulated metabolites such as MG (18:2), vaccenic acid, NAG, hypoxanthine, 1,2-DOPC, SHPC, 1-OPC, LysoPC, nicotinate, L-phenylalanine, tyramine, L-methionine, D-pipecolinic acid, and L-histidine were significantly increased (>2-fold) and clustered to form cluster 1, while cluster 2 consisted of the five down-regulated metabolites consisting of α-linolenic acid, D-ornithine, 2-hydroxyadenine, 2-methylbutyroyl carnitine, and GlcNAc in PiCB-treated mycelia of *P. digitatum* (Figure 4A). Similarly, the level of salicylic acid, palmitic acid, hypoxanthine, adenine, nicotinate, L-phenylalanine, L-tryptophan, L-methionine, L-valine, and L-serine dramatically increased after PiCB treatment in the negative mode (cluster 1, Figure 4B). On the contrary, the down-regulated metabolites of homogentisic acid, 9(S)-HODE, GDL, xanthosine, and sucrose in PiCB-treated mycelia of *P. digitatum* were grouped in cluster 2 (Figure 4B).

More focused studies are needed to find which mechanism is involved in differential metabolites having amino acids, lipids, fatty acids, tricarboxylic acid (TCA) cycle, purines, starch and sucrose were observed in both PiCB-treated and control mycelia (Table S1 and Figure 4). Amino acids are the important nutritional source for the *P. digitatum*, and their metabolic pathways may be targeted to check the fungal growth [33,34]. Among the 23 amino acids detected in *P. digitatum* mycelia, 18 amino acids were increased, while five amino acids were significantly decreased in PiCB-treated samples compared with the respective controls (Table S1 and Figure 4). The contents of L-phenylalanine, tyramine, L-tryptophan, L-leucine, betaine, L-methionine, dopamine, L-tyrosine, D-proline, L-pyroglutamic acid, L-arginine, L-aspartate, L-histidine, L-threonine, L-serine Glycyl-L-leucine, L-glutamine, and NAA were increased after 12 h in PiCB-treated *P. digitatum* compared to its control. In contrast, the levels of D-ornithine, L-glutamate, L-valine, argininosuccinic acid, and homogentisic acid were markedly decreased by 66.0, 32.4, 49.9, 21.6, and 55.1%, respectively, after 12 h in PiCB-treated mycelia compared with their controls. Present results reflected that the metabolic disorder of amino acids in *P. digitatum* mycelia was contributed to PiCB treatment. Similarly, carvacrol reported to suppress the mycelial growth of *P. digitatum* by interfering with amino acid metabolism [4].

Glycerophospholipids are an important class of lipids participating in multiple physiological processes in fungi [35], and their down regulations can significantly destroy the stability and permeability of cell membrane leading to the leakage of intracellular inclusions, promote lipid peroxidation following oxidative stress [36]. The results of current study showed that the lipid peroxidation in *P. digitatum* mycelia was associated with the increased levels of various acids (palmitic, pentadecanoic, stearidonic, and vaccenic acids) and the decreases in contents of linolenic, linoleic, and 16-hydroxypalmitic acids (Table S1 and Figure 4). This is in accordance with recent reports explaining that the cell apoptosis in the fungus are mainly caused by hydrolyzing the glycerophospholipids to elevate permeability of cell membranes under heavy oxidative stress [37].

While the contents of glycerophospholipids increased after PiCB treatment, the levels of unsaturated fatty acids significantly decreased (Table S1 and Figure 4). Regarding the metabolism of α-linolenic acid, the amounts of α-linolenic acid, linoleic acid, 9R, 10S-EpOME and 9(S)-HODE were decreased by 73.5%, 44.6%, 48.1%, and 47.5%, respectively, in PiCB-treated *P. digitatum* compared with control groups (Table S1). The current findings manifested that most of unsaturated fatty acids were oxidized and promoted programmed cell death (PCD) following PiCB exposure. Moreover, the current results also indicated that the production of saturated fatty acids in the PiCB-treated mycelia was significantly increased e.g. palmitic acid 1.44-fold and pentadecanoic acid 0.87-fold (S1). Clearly, the self-oxidation of unsaturated fatty acids that contributes to the mass production of saturated fatty acids, the degree of lipid peroxidation in the PiCB-treated *P. digitatum* was much higher. These results mimic a similar trend [38] where an essential oil of tea tree was tested against the gray mold phytopathogen of *Botrytis cinerea* to induce lipid peroxidation.

Moreover, the lower levels of unsaturated fatty acids in the fungal cells can bear the disadvantage of acetyl-CoA production, which essentially promotes the formation of succinate and provide energy for TCA cycle in the mitochondria [39]. As for the TCA cycle, the contents of citric acid and succinate were decreased significantly following PiCB treatment (Table S1 and Figure 4). This was possibly due to that citric acid and succinate that were greatly consumed in the PiCB-treated *P. digitatum* mitochondria. As a versatile precursor for fatty acid synthesis and as an energy source, both these fatty acids take part in cytoplasmic metabolism and energy metabolism in the mitochondria of *P. digitatum* mycelia were severely disrupted. Other studies reported a confirmed role of metabolites in respiration inside the mitochondria of some phytopathogens, including TCA cycle and ATP synthesis, could be disturbed by botanical fungicides such as citral, thymol, and salicylic acid [21,26].

It is interesting to notice that the contents of nicotinate and lactate were increased by PiCB treatment, since the two increased by approximately 2.51-fold and 1.37-fold in PiCB-treated mycelia (Table S1). It was suggested to induce more membrane peroxidation and lactate accumulation because of the effectiveness of PiCB to inhibit respiration. The observed increase in nicotinate and lactate levels are quiet obvious as many studies reported previously, which showed that a disorder of lactate metabolism in the fungal pathogens led to mass-produce lactate when treated with acetate owing to a membrane peroxidation involved in susceptibility or leading to cell death [40].

Similarly, the levels of uracil, hypoxanthine and xanthine were increased in PiCB-treated mycelia while the amount of adenosine was decreased compared with their respective controls. For instance, uracil experienced a 1.56-fold increase, hypoxanthine 3.27-fold increase, xanthine 1.94-fold increase, and adenosine 0.77-fold decrease (Table S1). In fungal pathogens, purines are known to be involved in nucleic acid synthesis, energy supply, amino acid anabolism, and stress tolerance [41]. The higher contents of uracil, hypoxanthine, and xanthine in the PiCB-treated group (Table S1 and Figure 4) might due to the oxidation of purine bases (e.g., adenine in DNA and guanine in RNA), suggesting that purines in *P. digitatum* mycelia were increased under the PiCB treatment because of the dissociation of nucleic acids.

The results displayed that the choline content was decreased by 23.7% in PiCB-treated *P. digitatum* compared to the control mycelia. Choline plays an important role as a regulatory factor for changing the phospholipid components of the cell membranes to avoid oxidative stress, and is known as the main precursor of betaine [42]. Interestingly, the betaine content was increased 1.65-fold in the PiCB-treated mycelia. Thus, the simultaneous decrease of choline and increase of betaine in the PiCB-treated samples suggest that the level of choline would probably be down-regulated by ROS efflux, which showed that the phospholipid components of cell membranes were destroyed after the PiCB treatment, leading to a membrane peroxidation in *P. digitatum* cells.

## 3. Materials and Methods

### 3.1. Isolation and Purification of Pinocembroside (PiCB)

PiCB (CAS-No. 75829-43-5, Figure 1) was isolated and purified from air-dried powder of hairy fig fruits (HFF) [14] by extraction applying an ultrasonic-associated method with 95% ethanol at reflux in the Jiangxi key laboratory for postharvest technology and nondestructive testing of fruits and vegetables at Jiangxi Agricultural University (Nanchang, China). The ethanol solution was evaporated under reduced pressure at 45 °C to recover the ethanol solvent. The ethanol extract was collected and subjected to D101 macro rein column chromatography, and eluted with different concentrations (0%, 30%, 50%, and 95%) of ethanol to afford four fractions (FHF1 to FHF4). The FHF4 was chromatographed on a column of Sephadex LH-20 eluted with methanol to afford eight fractions (FHF4a–FHF4h). Finally, the FHF4e was recrystallized with methanol to get PiCB.

### 3.2. Fungal Pathogen and Culture Conditions

*P. digitatum* (CGMCC 3.15410) was purchased from China General Microbiological Culture Collection Center (CGMCCC, Beijing, China), and embedded in 30% glycerol at –80 °C for its conservation. A pure culture of *P. digitatum* was activated on potato dextrose agar (PDA: 200 g peeled potatoes, 20 g glucose, 18 g agar powder and 1 L distilled water) medium at 25 ± 1 °C. After 7 d of culture, *P. digitatum* spore suspensions was collected, filtered, and adjusted to the desired concentration of 1.0 × 10^6^ CFU/mL with the aid of a Countess II FL automatic cell counter (Thermo Fisher Scientific, Waltham, MA, USA).

### 3.3. In Vitro Antifungal Activity Assay

#### 3.3.1. Mycelial Growth Assay

The method of Chen et al. [3] was employed to assay the in vitro antifungal activity of PiCB against *P. digitatum* with some modification. Briefly, the stock solution of PiCB was diluted with moderate amount of sterile 0.5% Tween 80 and mixed with PDA for obtaining the final concentrations of 0, 12.5, 25, 50, 100, 200, and 400 mg/L. A total of 15 μL of *P. digitatum* spore suspensions was injected into the Oxford cup (8 mm in diameter) placed in the center of each petri dish (90 mm in diameter). Finally, all plates were incubated at 25 °C for 7 d and measured the colony diameters of PiCB-treated and control groups using the cross method. Four replicates were used per treatment and the experiment was carried out at two separate times. The following formula was used for calculating the mycelial growth inhibition (MGI):

MGI (%) = [(Dc − Dt)/(Dc − 8)] × 100,

where Dc and Dt were the mean colony diameter of control and PiCB-treated sets, respectively.

The linear regression of the MGI under different PiCB treated concentrations (12.5–200 mg/L) was determined: *Y* = 0.3396*X* + 9.1243, *R*^2^ = 0.9682 (Figure 1). The half maximal effective concentration (EC_50_) value of PiCB against *P. digitatum* was 120.3 mg/L calculated based on the above linear regression.

#### 3.3.2. Measurement of Minimum Inhibitory Concentration (MIC) and Minimum Fungicidal Concentration (MFC)

The MIC and MFC were defined as the lowest concentration of PiCB with no visible fungal growth on a PDA plate after the second and sixth day of incubation at 25 °C, respectively [43].

#### 3.3.3. Determination of Mycelial Weights and Water-Retention Rate

The mycelial wet and dry weights of from PiCB-treated and control samples were determined as described by Wan et al. [4], and the unit of g/L used for representing the mycelial wet and dry weights of *P. digitatum*. The water-retention rate was calculated using the following equation:

Water-retention rate (%) = [(Wet weight − Dry weight)/Wet weight] × 100.

### 3.4. Microscopic Observations

The changes of PiCB on morphology and ultra-structure of *P. digitatum* were observed using both scanning electron microscope (SEM) and transmission electron microscope (TEM). PiCB treatment and sample preparation were followed the method previously reported by Xin et al. [7]. Finally, the PiCB-treated and control samples were observed using SEM (JSM-6360LV, JEOL Ltd., Tokyo, Japan) operated at 2500× of magnification, and TEM (JEM-1400, JEOL Ltd., Tokyo, Japan) operated at 20,000× of magnification, respectively.

### 3.5. Preparation of Metabolite Samples

30 μL of *P. digitatum* spores was added to 150 mL PDB and incubated in an incubator shaker (IS-RDV3, Crystal Technology & Industries, Inc., Dallas, America) at 160 rpm for 48 h at 25 °C. For intracellular metabolite samples, about 2.0 g of *P. digitatum* mycelia was collected and washed three times with sterile double-distilled water and re-suspended in a sterilized conical flask containing PiCB solution at 100 mg/L (1/2 MIC) at 160 rpm, and the mycelia treated without PiCB was used as control. The PiCB-treated and control mycelia were obtained after shaking culture of 12 h at 25 °C, placed in a vacuum freeze drier (FD-1000, Tokoy, Japan) for 24 h, and kept at −80 °C for metabolomics analysis.

The freeze-dried samples (80 mg) was mixed with 20 μL of ultrapure water and homogenized for 60 s on a FastPrep-24 tissue homogenizer (MP Biomedicals, Santa Ana, CA, USA), and added 80 μL of methanol/acetonitrile (2:1, *v/v*). After homogenizing for 60 s, the solution was ultrasonic sonic disrupted for 30 min twice and placed in −20 °C condition for 1 h. The supernate collected after centrifugation (14,000 rpm for 10 min at 4 °C) was freeze-dried and stored at −80 °C for next detection.

### 3.6. UHPLC-Q-TOF/MS Assays

The analytes were separated using a hydrophilic interaction liquid chromatography (HILIC) column (100 mm × 3.1 mm × 1.7 μm) and detected by means of an Agilent 1290 Infinity LC Ultra-high pressure liquid chromatography (UHPLC, Agilent Technologies, Santa Clara, CA, USA) system, which was coupled to a quadrupole time-of-flight (AB Triple TOF 5600, AB Sciex, CA, USA) mass spectrometer equipped with an electron spray ionization as positive (ESI+) and negative (ESI–) modes. The ESI source conditions were: Ion Source Gas1 (Gas1) as 60, Ion Source Gas2 (Gas2) as 60, curtain gas (CUR) as 30, source temperature: 600 °C, Ion Spray Voltage Floating (ISVF) ± 5500 V. The *m/z* range and accumulation time in TOF MS scan were set as 60–1000 Da and 0.20 s/spectrum, while above Two parameters in product ion scan were set as 25–1000 Da and 0.50 s/spectrum, respectively. Secondary ion mass-spectrometry was obtained by an information dependent acquisition (IDA) under the high sensitivity mode. The declustering potential (DP) was ± 60 V (positive and negative modes), and collision energy was 35 ± 15 eV.

### 3.7. Statistical Analysis

The raw data was converted to MzXML format by mean of ProteoWizard and conducted using R package XCMS. The Metabolites constructions were identified by matching to the molecular weight (<25 ppm) and secondary spectrum diagram in our standards database.

## 4. Conclusions

In summary, a flavonone PiCB isolated from *Ficus hirta* Vahl. fruit was demonstrated to exert a significant inhibitory effect on *P. digitatum* mycelial growth, and its antifungal mechanisms of membrane peroxidation might be linked with the disruption of the amino acid metabolism, lipid metabolism, fatty acid metabolism, TCA cycle, and purine metabolism. The study displays the potential of PiCB as a novel antifungal agent for controlling postharvest fungal decay in citrus fruit and emerges as a promising candidate for citrus preservatives.

## Figures and Tables

**Table 1:** The mycelial weights and water-retention of *P. digitatum* treated with different concentrations of PiCB.

Concentrations (mg/L)	Mycelial Weight (g/L)		Water-Retention Rate (%)
	Wet Weight	Dry Weight	
0	34.32 ± 1.588 a	3.82 ± 0.224 a	88.9 ± 0.14 a
12.5	32.73 ± 2.223 a	3.68 ± 0.127 a	88.7 ± 0.38 a
25	27.13 ± 1.446 b	3.40 ± 0.042 b	87.3 ± 0.43 b
50	22.37 ± 1.595 c	3.32 ± 0.076 bc	85.4 ± 0.69 c
100	20.14 ± 0.884 d	3.14 ± 0.058 cd	84.5 ± 0.40 c
200	18.88 ± 0.527 d	3.07 ± 0.066 d	83.4 ± 0.21 d
400	18.23 ± 0.388 d	3.03 ± 0.069 d	83.3 ± 0.13 d

Values are mean ± S.E. The data followed by different letters (a, b, c and d) within the column are significantly different according to Duncan’s test (*p* < 0.05).

## Supplementary Materials

The following are available online at https://www.mdpi.com/2223-7747/9/1/17/s1, Figure S1. A volcano plot of all metabolite ions.; Table S1. Metabolites identified by UPLC-Q-TOF-MS of *Penicillium digitatum* mycelia treated with pinocembroside (PiCB).
