# Supplementary material for: UHPLC-Q-TOF/MS-Based Metabolomics Approach Reveals the Antifungal Potential of Pinocembroside against Citrus Green Mold Phytopathogen
Source: Plants (Basel). 2019 Dec 22;9(1):17. doi: 10.3390/plants9010017 (PMC7020183; doi:10.3390/plants9010017)
Supplement: Supplementary file 1 [file plants-09-00017-s001.pdf]

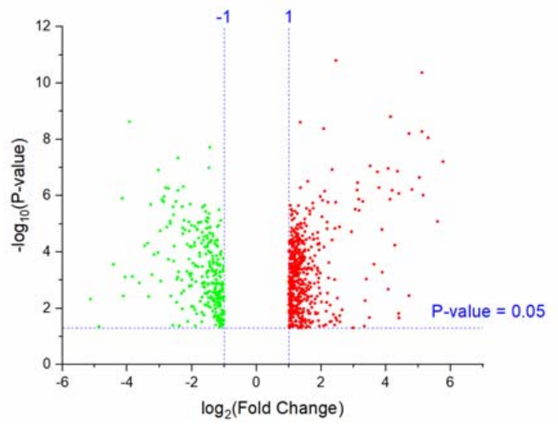

A

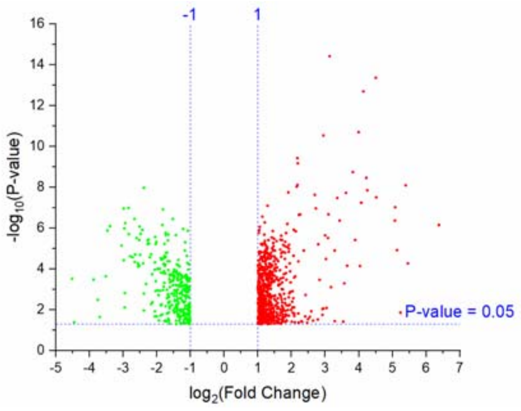

B

Figure S1: A vulcano plot of all metabolite ions detected by negative mode (**A**) and positive mode (**B**).

Table S1 metabolites identified by UPLC-Q-TOF-MS of *P. digitatum* mycelia treated with PiCB

Ion mode	Adduct	RT (min)	Metabolite [a]	Detected m/z	Molecular formula	Theoretical m/z	Log_2-FC [b]	Functional category
ESI(+)	(M+H)+	0.78	α-Linolenic acid	279.23104	$C_{18}H_{32}O_2$	280.24023	-1.92	Fatty acids metabolism
	(M+H-H_2O)+	1.20	MG(18:2)	337.27287	$C_{21}H_{38}O_4$	354.52402	2.32	Lipid metabolism
	(M+Na)+	1.27	16-Hydroxypalmitic acid	295.22591	$C_{16}H_{32}O_3$	272.42899	-0.26	Fatty acids metabolism
	(M+Na)+	1.33	PC(16:0/16:0)	756.55382	$C_{40}H_{80}NO_8P$	733.56213	0.74	Lipid metabolism
	(M+H)+	1.48	Nicotinamide	123.05374	$C_6H_6N_2O$	122.04801	-0.44	Vitamin metabolism
	(M+H-H_2O)+	2.51	Adenosine	250.09251	$C_{10}H_{13}N_5O_4$	267.09674	0.77	Purine metabolism
	(M+H)+	3.38	Pyridoxine	170.07964	$C_8H_{11}NO_3$	169.07388	-0.72	Vitamin metabolism
	(M+H)+	3.65	Stearidonic acid	277.21523	$C_{18}H_{28}O_2$	276.20892	0.68	Fatty acids metabolism
	(M+H)+	3.97	4-Aminobenzoate	138.05324	$C_7H_7NO_2$	137.04768	-0.69	Folate biosynthesis
	(M+H)+	4.56	Vaccenic acid	251.09068	$C_{18}H_{34}O_2$	282.25589	1.12	Fatty acids metabolism
	M+	4.78	Choline	104.10542	$C_5H_{14}NO$	104.10754	-0.39	Lipid metabolism
	(M+H)+	5.29	Uracil	113.03290	$C_4H_4N_2O_2$	112.02728	0.64	Nucleic metabolism
	(M+H-H2O)+	5.57	NAG	204.08526	$C_8H_{15}NO_6$	221.08990	2.23	Amino sugar and nucleotide sugar metabolism
	(M+H)+	5.61	Hypoxanthine	137.04440	$C_5H_4N_4O$	136.03851	1.24	Purine metabolism
	(2M+H)+	5.86	2'-O-Methyluridine	515.20869	$C_{10}H_{15}N_3O_5$	257.10117	0.96	Nucleic metabolism
	(M-H+2Na)+	5.99	SOPC	832.57082	$C_{44}H_{86}NO_8P$	788.14697	0.66	Lipid metabolism
	(M+Na)+	6.04	DOPC	808.57063	$C_{44}H_{84}NO_8P$	786.13098	1.22	Lipid metabolism
	(M+H)+	6.09	SHPC	524.36908	$C_{26}H_{54}NO_7P$	523.36377	1.02	Lipid metabolism
	M+	6.27	PAF 18:1	550.38505	$C_{28}H_{56}NO_7P$	549.37946	0.68	Lipid metabolism
	(M+H)+	6.39	1-OPC	522.35446	$C_{26}H_{52}NO_7P$	521.67798	1.51	Lipid metabolism
	(M+H)+	6.63	DMPC	468.30743	$C_{22}H_{46}NO_7P$	467.30118	0.72	Lipid metabolism

(M+Na)+	6.78	LysoPC(16:0)	518.32244	$C_{24}H_{50}NO_7P$	495.64001	1.18	Lipid metabolism
(M+H-H2O)+	6.84	D-Ornithine	115.08481	$C_{44}H_{84}NO_8P$	786.13098	-1.56	Amino acid metabolism
(M+H)+	6.94	LPE 16:0	454.29271	$C_{21}H_{44}NO_7P$	453.28549	0.29	Lipid metabolism
(M+H)+	7.17	Xanthine	153.03889	$C_5H_4N_4O_2$	152.03343	0.96	Purine metabolism
(M+H)+	7.32	Nicotinate	124.03761	$C_6H_5NO_2$	123.03203	1.33	Vitamin metabolism
(M+H)+	7.65	2-Hydroxyadenine	152.05523	$C_5H_5N_5O$	151.12900	-1.59	Purine metabolism
M+	8.30	2-Methylbutyroyl carnitine	246.16910	$C_{12}H_{23}NO_4$	245.32808	-1.23	Lipid metabolism
(M+H)+	8.68	L-Phenylalanine	166.08464	$C_9H_{11}NO_2$	165.07899	1.21	Amino acid metabolism
(M+H-H2O)+	8.70	Tyramine	120.07916	$C_8H_{11}NO$	137.08406	1.47	Amino acid metabolism
(M+H)+	8.74	L-Tryptophan	205.09561	$C_{11}H_{12}N_2O_2$	204.08990	0.76	Amino acid metabolism
(M+H-H2O)+	8.74	Indole-3-lactic acid	188.06937	$C_{11}H_{11}NO_3$	205.07388	0.92	IAA biosynthesis
(M+H)+	8.90	L-Leucine	132.10048	$C_6H_{13}NO_2$	131.09464	0.74	Amino acid metabolism
(M+H)+	9.20	Betaine	118.08475	$C_5H_{11}NO_2$	117.07898	0.72	Amino acid metabolism
(M+H)+	9.56	L-Methionine	150.05697	$C_5H_{11}NO_2S$	149.05106	1.21	Amino acid metabolism
(M+H)+	10.09	L-Tyrosine	182.07994	$C_9H_{11}NO_3$	181.07390	0.95	Amino acid metabolism
(M+H-H2O)+	10.10	Dopamine	136.07385	$C_8H_{11}NO_2$	153.07898	0.80	Amino acid metabolism
(M+H)+	10.35	D-Pipecolinic acid	130.08457	$C_6H_{11}NO_2$	129.07898	1.08	Piperidine biosynthesis
(M+H)+	10.47	D-Proline	116.06883	$C_5H_9NO_2$	115.06333	0.32	Amino acid metabolism
(M+H)+	11.67	GlcNAc	425.17532	$C_{16}H_{28}N_2O_{11}$	424.42402	-0.86	Amino sugar and nucleotide sugar metabolism
(M+NH4)+	12.26	Sucrose	360.14923	$C_{12}H_{22}O_{11}$	342.11621	-0.65	Starch and sucrose metabolism
(M+H)+	12.30	GDL	179.05367	$C_6H_{10}O_6$	178.14000	-0.55	Pentose phosphate pathway
(M+NH4)+	12.40	L-Pyroglutamic acid	147.07473	$C_5H_7NO_3$	129.04259	0.72	Amino acid metabolism
M+	12.98	Glycerophosphocholine	258.10868	$C_8H_{20}NO_6P$	257.22124	0.24	Lipid metabolism

	(M+H)+	13.11	L-Glutamate	148.05890	$C_5H_9NO_4$	147.05316	-0.56	Amino acid metabolism
	(M+H)+	13.28	L-Aspartate	134.04320	$C_4H_7NO_4$	133.03751	0.98	Amino acid metabolism
	(M+H)+	13.80	L-Histidine	156.07521	$C_6H_9N_3O_2$	155.06947	1.59	Amino acid metabolism
	(M+H)+	14.25	D-Maltose	343.12237	$C_{12}H_{22}O_{11}$	342.29700	-0.73	Starch and sucrose metabolism
	(M+H)+	14.42	Argininosuccinic acid	291.12903	$C_{10}H_{18}N_4O_6$	290.12262	-0.35	Amino acid metabolism
	(M+H)+	14.46	UMP	325.04215	$C_9H_{11}N_2O_9P$	324.03586	-0.68	Pyrimidine metabolism
	(M+NH$_4$)+	14.71	Maltotriose	522.20204	$C_{18}H_{32}O_{16}$	504.16904	0.64	Carbohydrate metabolism
	(M+H)+	17.07	L-Arginine	175.11737	$C_6H_{14}N_4O_2$	174.11168	0.67	Amino acid metabolism
ESI(-)	(M-H)-	0.93	Salicylic acid	137.02373	$C_7H_6O_3$	138.03169	2.24	Phenylalanine metabolism
	(M-H)-	1.22	α-Linolenic acid	277.21631	$C_{18}H_{30}O_2$	278.22461	-0.71	Fatty acids metabolism
	(M-H)-	1.23	Pentadecanoic acid	241.21642	$C_{15}H_{30}O_2$	242.22459	0.87	Fatty acids metabolism
	(M-H)-	1.93	9R,10S-EpOME	295.22724	$C_{18}H_{32}O_3$	296.23514	-0.95	Fatty acids metabolism
	(M-H$_2$O-H)-	2.16	D-Glucose	161.04505	$C_6H_{12}O_6$	180.06340	-0.28	Glycolysis/gluconeogenesis
	(M-H)-	2.31	Homogentisic acid	167.03472	$C_8H_8O_4$	168.04227	-1.16	Amino acid metabolism
	(M-H)-	2.38	2'-O-Methyluridine	257.07717	$C_{10}H_{14}N_2O_6$	258.08521	0.75	Nucleic metabolism
	(M-H)-	3.09	9(S)-HODE	295.22697	$C_{18}H_{32}O_3$	296.23514	-0.93	Fatty acids metabolism
	(M-H)-	3.92	GDL	177.04001	$C_6H_{10}O_6$	178.14000	-1.77	Pentose phosphate pathway
	(M+K-2H)-	4.22	5'-O-Methylthymidine	293.05383	$C_{11}H_{16}N_2O_5$	256.10593	0.97	Nucleic metabolism
	(M-H)-	4.60	Palmitic acid	255.23218	$C_{16}H_{32}O_2$	256.24023	1.44	Fatty acids metabolism
	(M-H)-	4.76	Uridine	243.06223	$C_9H_{12}N_2O_6$	244.06953	0.48	Pyrimidine metabolism
	(M-H)-	4.77	Uracil	111.01938	$C_4H_4N_2O_2$	112.02728	0.33	Nucleic metabolism
	(M-H)-	4.99	Hypoxanthine	135.03067	$C_5H_4N_4O_2$	152.03343	1.71	Purine metabolism
	(M-H)-	5.08	Linoleic acid	279.23229	$C_{18}H_{32}O_2$	280.24023	-0.85	Fatty acids metabolism
	(M-H)-	5.14	D-Threitol	121.05012	$C_4H_{10}O_4$	122.12168	-0.26	Carbohydrate metabolism
	(M-H)-	5.52	Adenine	134.04680	$C_5H_5N_5$	135.05450	1.03	Purine metabolism

(M-H)-	5.94	LPE 16:0	452.27664	$C_{21}H_{44}NO_7P$	453.28549	0.47	Lipid metabolism
(M-H)-	6.40	Nicotinate	122.02412	$C_6H_5NO_2$	123.03203	1.05	Vitamin metabolism
(M-H)-	6.44	Xanthine	151.02594	$C_5H_4N_4O_2$	152.03343	0.57	Purine metabolism
(M-H)-	6.71	DL-lactate	89.02403	$C_3H_6O_3$	90.03169	0.45	Fatty acids metabolism
(M-H)-	7.68	L-Phenylalanine	164.07149	$C_9H_{11}NO_2$	165.07899	1.56	Amino acid metabolism
(M-H)-	7.72	L-Tryptophan	203.08214	$C_{11}H_{12}N_2O_2$	204.08990	1.37	Amino acid metabolism
(M-H)-	8.49	Glycyl-L-leucine	187.10830	$C_8H_{16}N_2O_3$	188.22701	0.97	Amino acid metabolism
(M-H)-	8.54	L-Methionine	148.04340	$C_5H_{11}NO_2S$	149.05106	1.13	Amino acid metabolism
(M+CH₃COO)-	9.13	Dulcitol	241.09260	$C_6H_{14}O_6$	182.07904	1.03	Galactose metabolism
(M-H)-	9.15	L-Valine	116.07110	$C_5H_{11}NO_2$	117.07898	-1.00	Amino acid metabolism
(M-H)-	9.26	Xanthosine	283.06733	$C_{10}H_{12}N_4O_6$	284.07571	0.60	Purine metabolism
(M-H)-	10.68	L-Threonine	118.05041	C4H9NO3	119.05824	0.55	Amino acid metabolism
(M+CH₃COO)-	11.26	Galactinol	401.12825	C12H22O11	342.11621	-0.67	Galactose metabolism
(M-H)-	11.27	Sucrose	341.10786	$C_{12}H_{22}O_{11}$	342.11621	-1.32	Starch and sucrose metabolism
(M-H)-	11.36	Dihydrothymine	127.05061	$C_5H_8N_2O_2$	128.13170	0.57	Nucleic metabolism
(M-H)-	11.37	L-Glutamine	145.06137	$C_5H_{10}N_2O_3$	146.06914	0.69	Amino acid metabolism
(M-H)-	11.43	L-Serine	104.03465	$C_3H_7NO_3$	105.04259	1.22	Amino acid metabolism
(M-H)-	11.60	Succinate	117.01909	$C_4H_6O_4$	118.02661	-0.45	TCA cycle
(M-H)-	11.61	NAG	188.05598	$C_7H_{11}NO_5$	189.06372	-0.50	Amino sugar and nucleotide sugar metabolism
(M-H)-	11.70	L-Histidine	154.06175	$C_6H_9N_3O_2$	155.06947	0.98	Amino acid metabolism
(M-H)-	11.79	NAA	174.04040	$C_6H_9NO_5$	175.04807	0.74	Amino acid metabolism
(M-H)-	11.96	L-Glutamate	146.04563	$C_5H_9NO_4$	147.05316	-0.50	Amino acid metabolism
(M-H)-	12.03	L-Malic acid	133.01385	$C_4H_6O_5$	134.02151	0.18	TCA cycle
(M-H)-	12.14	L-Aspartate	132.02967	$C_4H_7NO_4$	133.03751	0.78	Amino acid metabolism

(M-H)-	12.60	Glucosamine-1-p	258.03788	$C_6H_{14}NO_8P$	259.15102	-0.80	Glycolysis/gluconeogenesis
(M-H)-	12.66	Maltitol	343.12327	$C_{12}H_{24}O_{11}$	344.13187	0.38	Carbohydrate metabolism
(M-H)-	13.00	UDP-GlcNAc	606.07275	$C_{17}H_{27}N_3O_{17}P_2$	607.08154	0.53	Glycolysis/gluconeogenesis
(M-H)-	13.58	Galactonic acid	195.05072	$C_6H_{12}O_7$	196.15500	-0.61	Galactose metabolism
(M-H)-	13.76	Glucose 1-p	259.02208	$C_6H_{13}O_{10}P$	276.13518	-0.73	Glycolysis/gluconeogenesis
(M+K-2H)-	13.81	Acetyl phosphate	176.93580	$C_2H_5O_5P$	139.98746	0.58	Pyruvate metabolism
(M-H)-	14.04	Maltotriose	503.15976	$C_{18}H_{32}O_{16}$	504.16904	0.81	Carbohydrate metabolism
(M-H)-	15.24	Citrate acid	191.01956	$C_6H_8O_7$	192.02699	-0.66	TCA cycle
(M-H)-	16.12	L-Arginine	173.10436	$C_6H_{14}N_4O_2$	174.20399	0.61	Amino acid metabolism

[a] Abbreviations: MG, 1-Linoleoyl-rac-glycerol; PC, phosphotidylcholine; NAG, N-Acetyl-D-glucosamine; SOPC, 1-Stearoyl-2-oleoyl-sn-glycerol 3-phosphocholine; DOPC, 1,2-dioleoyl-sn-glycero-3-phosphatidylcholine; SHPC, 1-Stearoyl-sn-glycerol 3-phosphocholine; PAF 18:1, 1-O-(cis-9-Octadecenyl)-2-O-acetyl-sn-glycero-3-phosphocholine; 1-OPC, 1-oleoyl-sn-glycero-3-phosphocholine; DMPC, 1-Myristoyl-sn-glycero-3-phosphocholine; LysoPC(16:0), 1-Palmitoyl-sn-glycero-3-phosphocholine; GlcNAc, N,N'-Diacetylchitobiose; LPE 16:0, 1-Palmitoyl-2-hydroxy-sn-glycero-3-phosphoethanolamine; GDL, D-Glucono-1,5-lactone; UMP, Uridine 5'-monophosphate; NAA, N-Acetyl-L-aspartic acid; UDP-GlcNAc, UDP-N-acetyl-glucosamine; Glucosamine-1-p, D-Glucosamine 1-phosphate; Glucose -1-p, D-Glucose 1-phosphate.

[b] FC: fold change, PiCB treatment VS control group.

Figure S2. Mass spectrum of marker metabolites

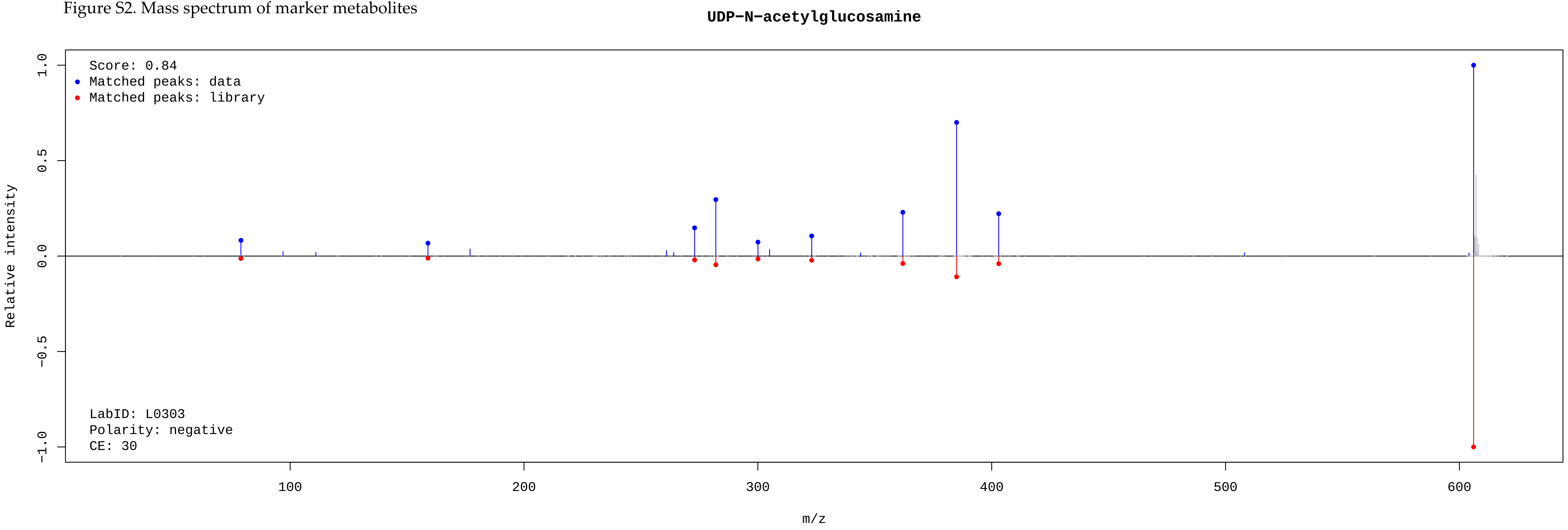

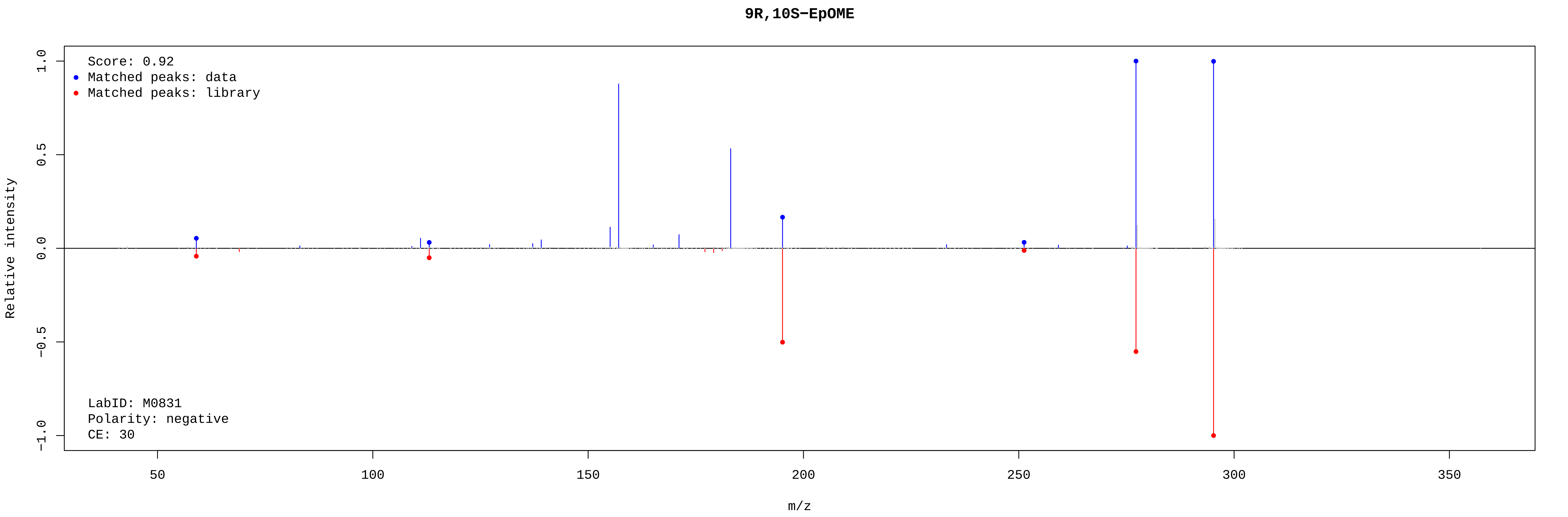

9R,10S−EpOME
Score: 0.92
Matched peaks: data
Matched peaks: library
LabID: M0831
Polarity: negative
CE: 30
Relative intensity
1.0
0.5
0.0
−0.5
−1.0
50
100
150
200
250
300
350
m/z

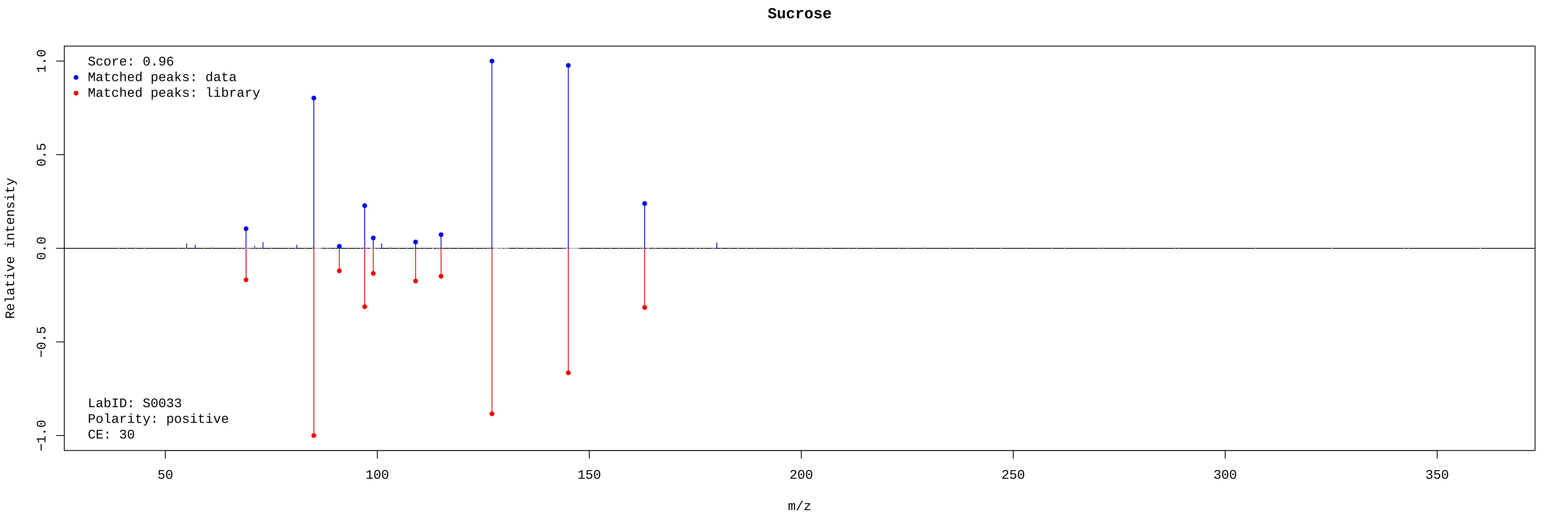
Sucrose
Score: 0.96
Matched peaks: data
Matched peaks: library
LabID: S0033
Polarity: positive
CE: 30
Relative intensity
m/z
1.0
0.5
0.0
−0.5
−1.0
50
100
150
200
250
300
350

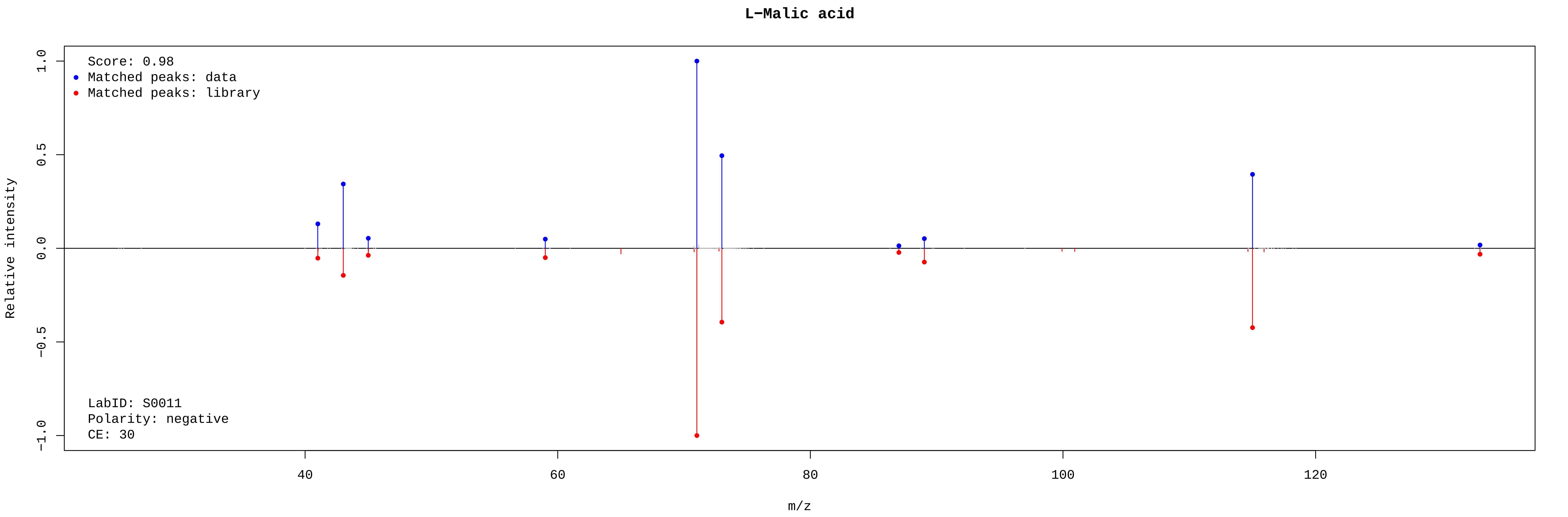

L−Malic acid
Score: 0.98
Matched peaks: data
Matched peaks: library
LabID: S0011
Polarity: negative
CE: 30
Relative intensity
m/z
1.0
0.5
0.0
−0.5
−1.0
40
60
80
100
120

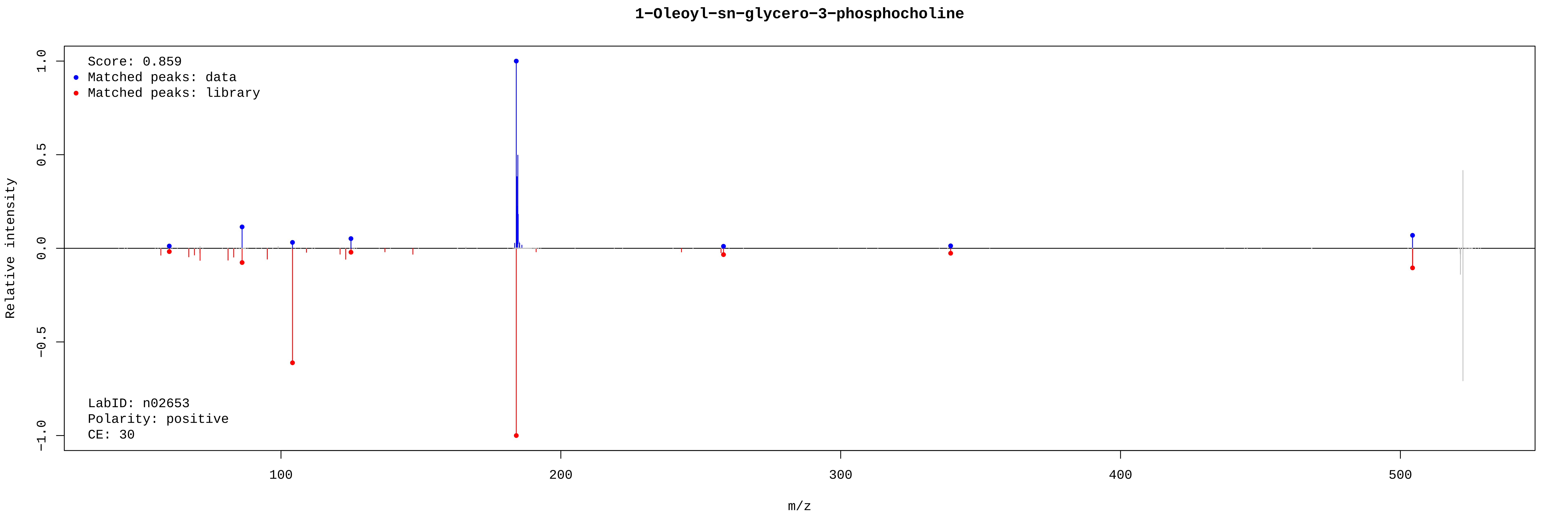
1-Oleoyl-sn-glycero-3-phosphocholine
Score: 0.859
Matched peaks: data
Matched peaks: library
LabID: n02653
Polarity: positive
CE: 30
Relative intensity
1.0
0.5
0.0
-0.5
-1.0
100
200
300
400
500
m/z

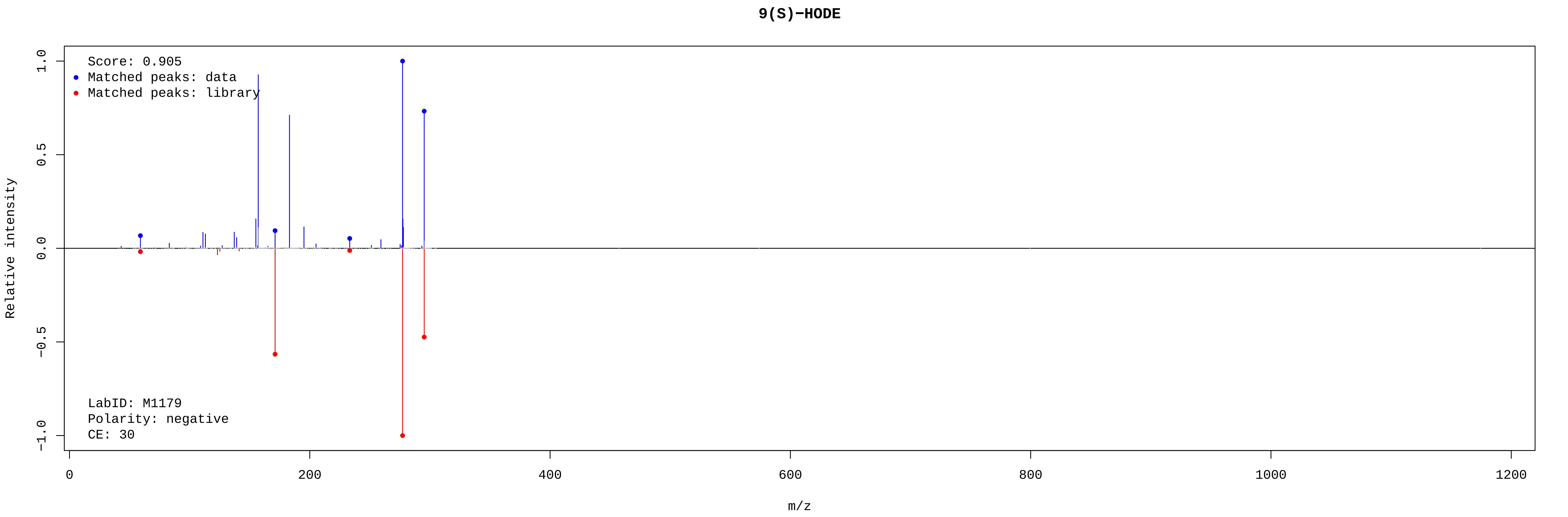

9(S)−HODE
Score: 0.905
Matched peaks: data
Matched peaks: library
LabID: M1179
Polarity: negative
CE: 30
Relative intensity
m/z
1.0
0.5
0.0
−0.5
−1.0
0
200
400
600
800
1000
1200

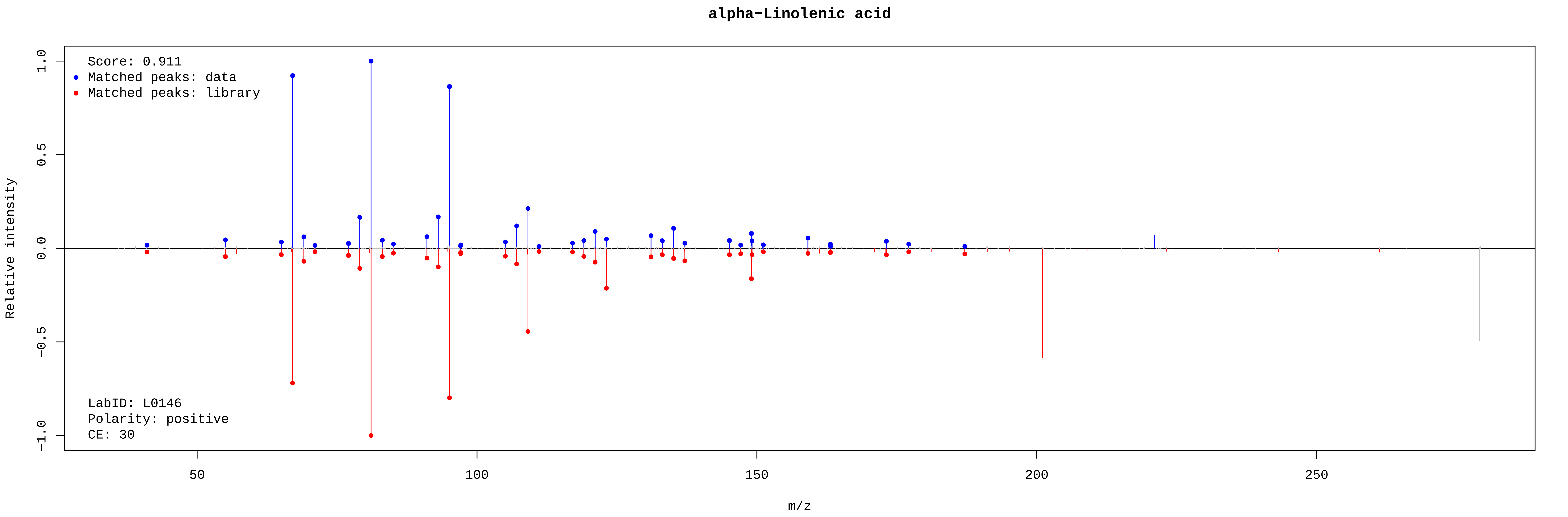
alpha−Linolenic acid
Score: 0.911
Matched peaks: data
Matched peaks: library
LabID: L0146
Polarity: positive
CE: 30
Relative intensity
1.0
0.5
0.0
−0.5
−1.0
50
100
150
200
250
m/z

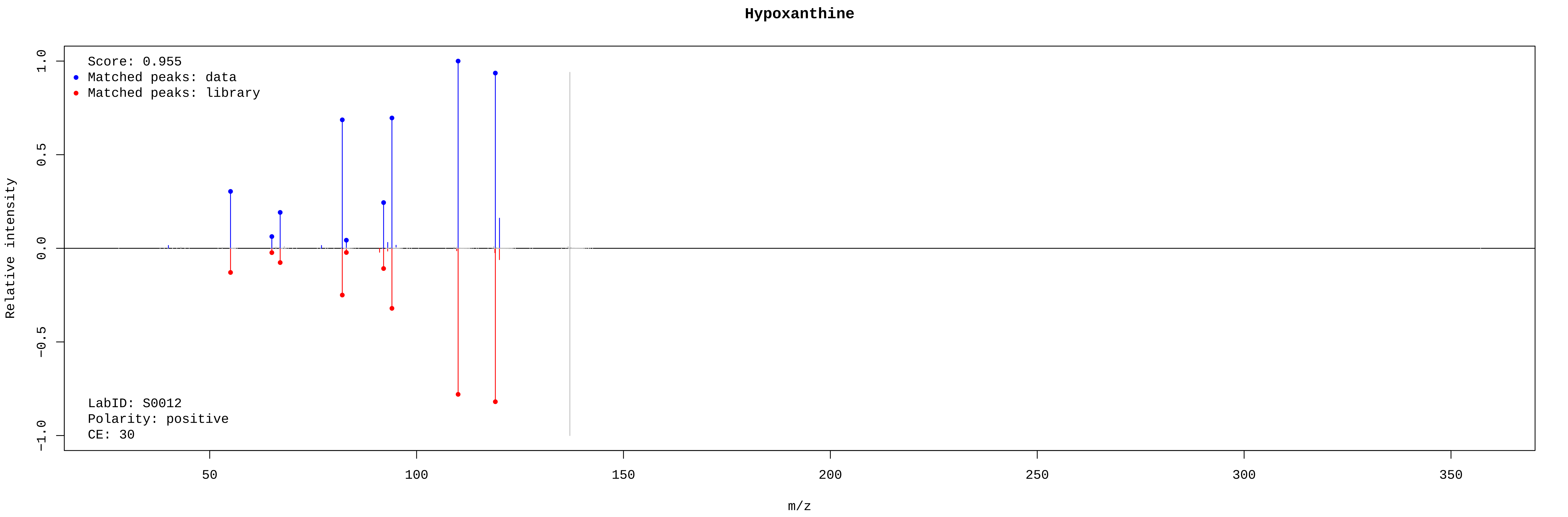
Hypoxanthine
Score: 0.955
Matched peaks: data
Matched peaks: library
LabID: S0012
Polarity: positive
CE: 30
Relative intensity
1.0
0.5
0.0
−0.5
−1.0
50
100
150
200
250
300
350
m/z

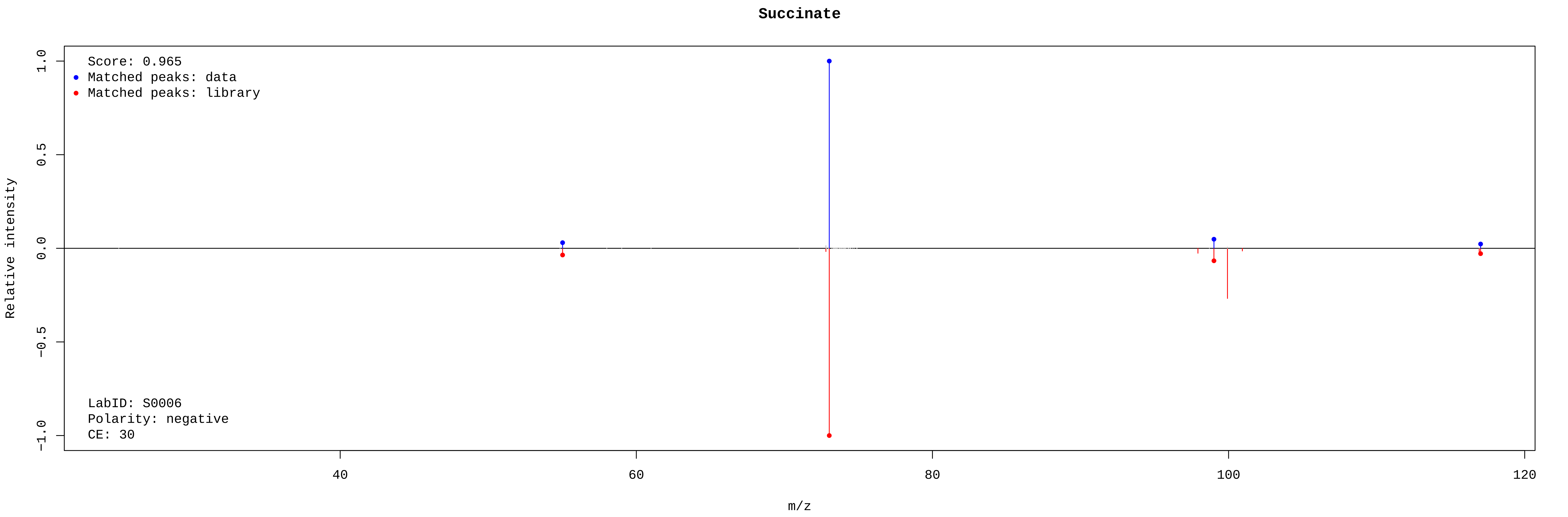

Succinate
Score: 0.965
Matched peaks: data
Matched peaks: library
LabID: S0006
Polarity: negative
CE: 30
Relative intensity
1.0
0.5
0.0
-0.5
-1.0
40
60
80
100
120
m/z

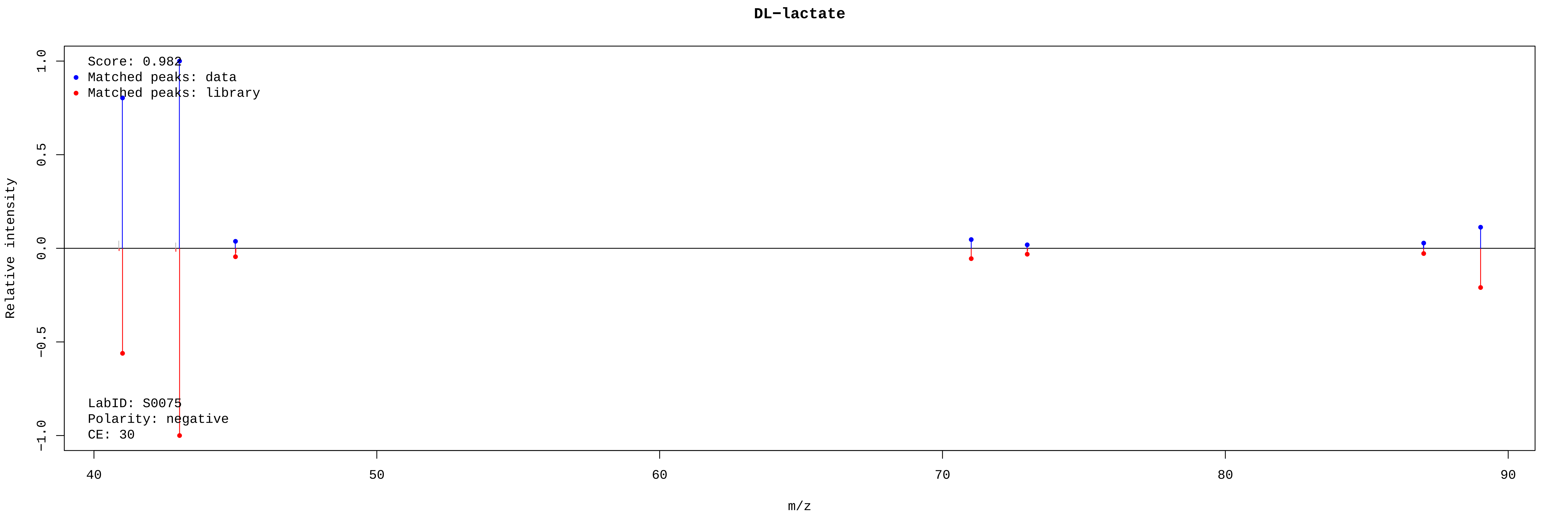
DL-lactate
Score: 0.982
Matched peaks: data
Matched peaks: library
LabID: S0075
Polarity: negative
CE: 30
Relative intensity
1.0
0.5
0.0
-0.5
-1.0
40
50
60
70
80
90
m/z

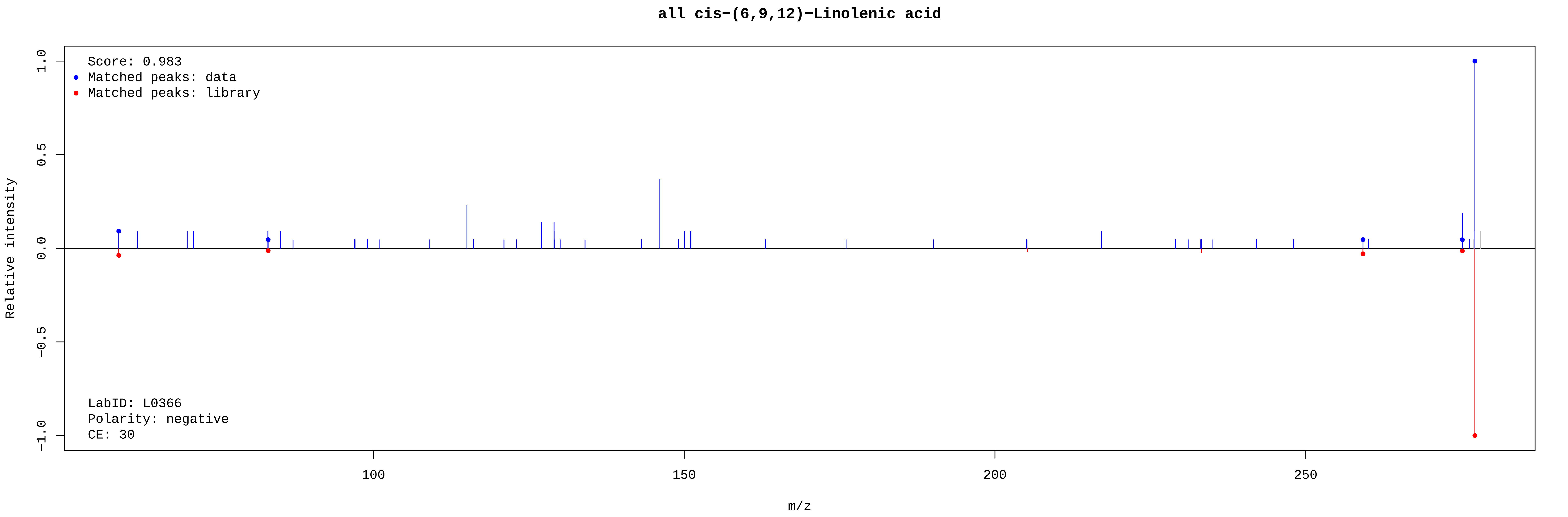

all cis-(6,9,12)-Linolenic acid
Score: 0.983
Matched peaks: data
Matched peaks: library
LabID: L0366
Polarity: negative
CE: 30
Relative intensity
1.0
0.5
0.0
-0.5
-1.0
100
150
200
250
m/z

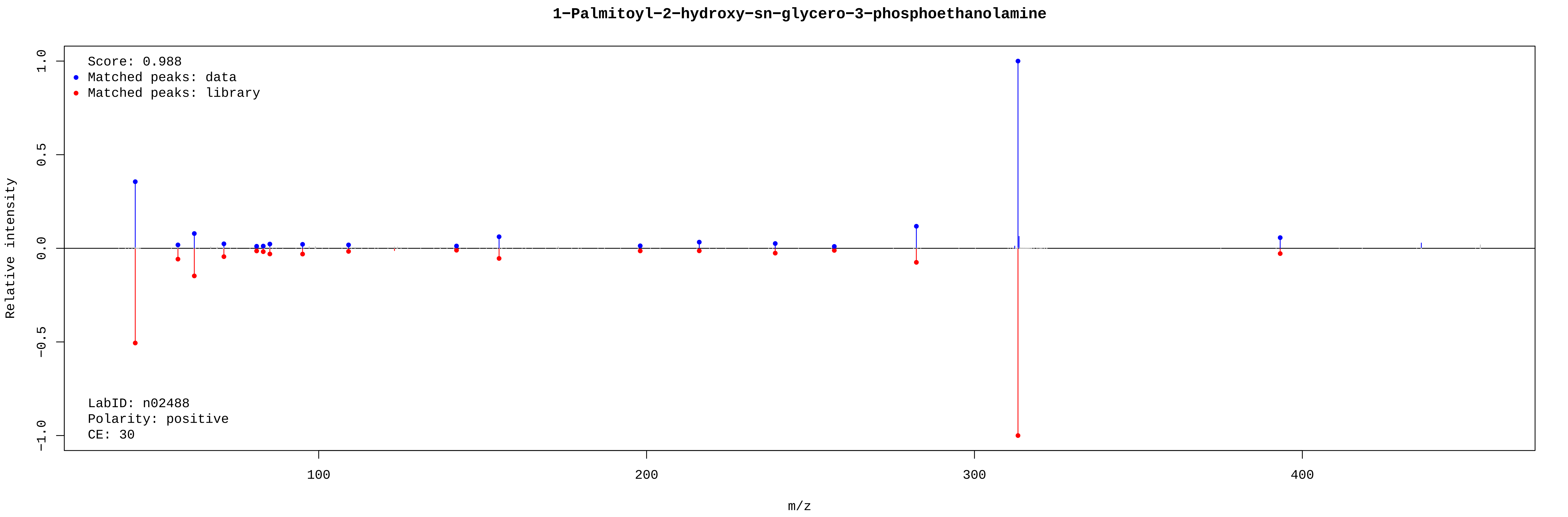

1-Palmitoyl-2-hydroxy-sn-glycero-3-phosphoethanolamine
Score: 0.988
Matched peaks: data
Matched peaks: library
LabID: n02488
Polarity: positive
CE: 30
Relative intensity
1.0
0.5
0.0
-0.5
-1.0
100
200
300
400
m/z

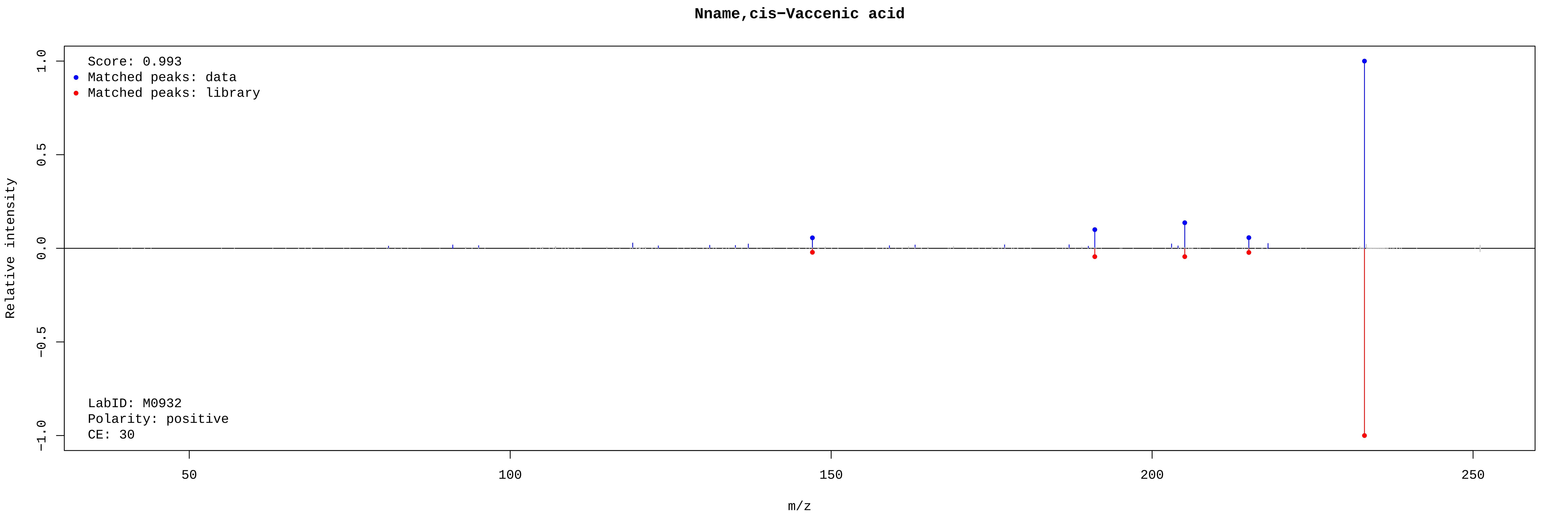

Nname,cis−Vaccenic acid
Score: 0.993
Matched peaks: data
Matched peaks: library
LabID: M0932
Polarity: positive
CE: 30
Relative intensity
m/z
1.0
0.5
0.0
−0.5
−1.0
50
100
150
200
250

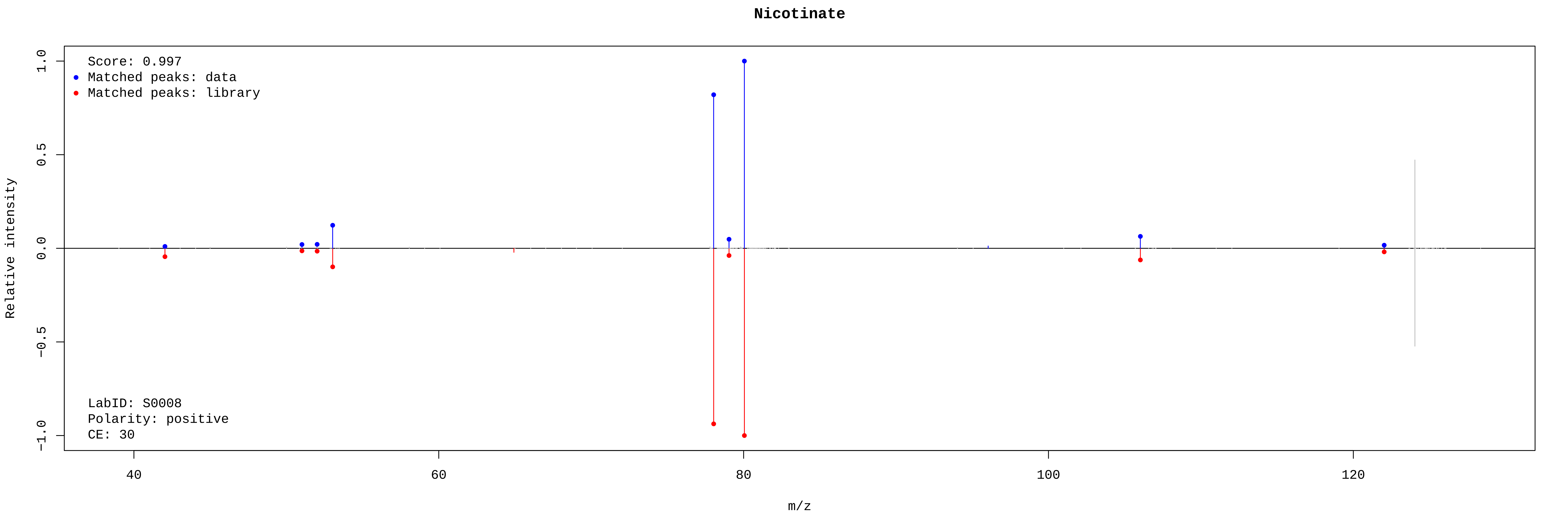
Nicotinate
Score: 0.997
Matched peaks: data
Matched peaks: library
LabID: S0008
Polarity: positive
CE: 30
Relative intensity
1.0
0.5
0.0
−0.5
−1.0
m/z
40
60
80
100
120

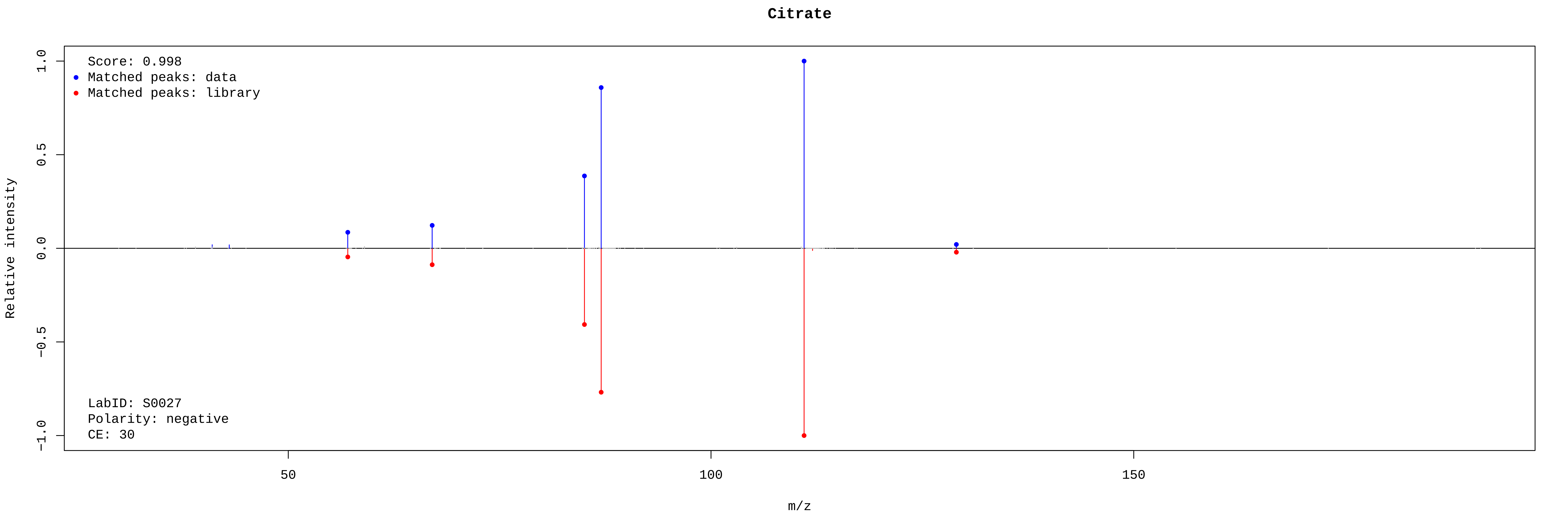
Citrate
Score: 0.998
Matched peaks: data
Matched peaks: library
LabID: S0027
Polarity: negative
CE: 30
Relative intensity
m/z
1.0
0.5
0.0
−0.5
−1.0
50
100
150

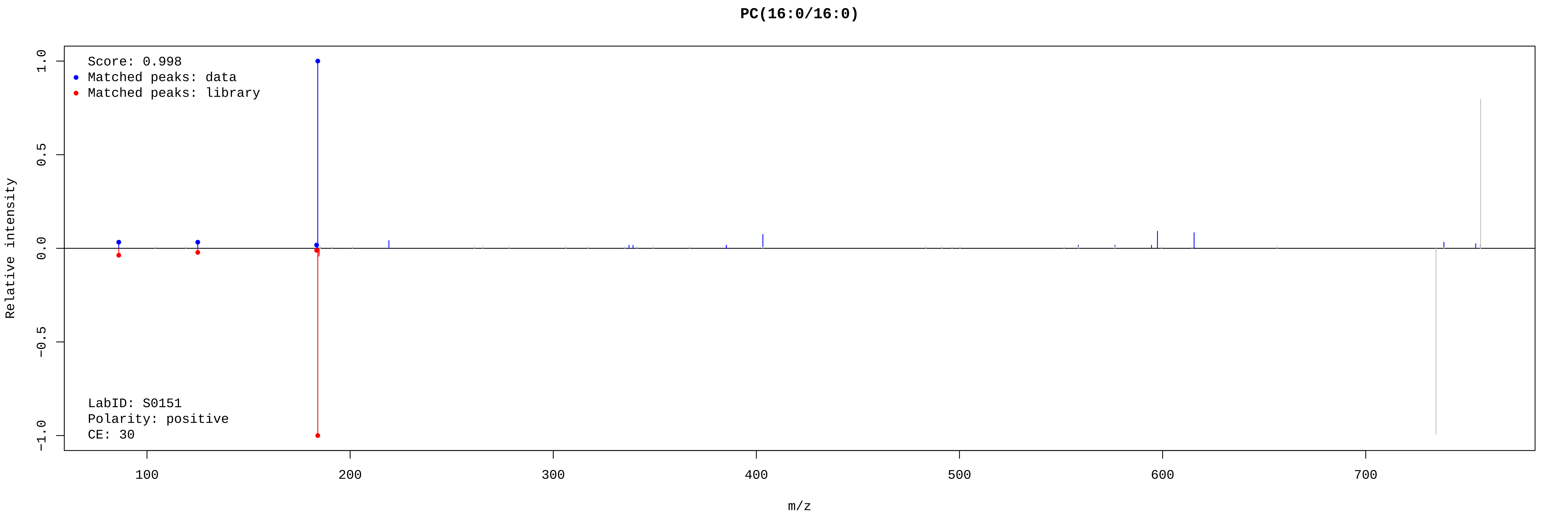
PC(16:0/16:0)
Score: 0.998
Matched peaks: data
Matched peaks: library
LabID: S0151
Polarity: positive
CE: 30
Relative intensity
m/z
1.0
0.5
0.0
−0.5
−1.0
100
200
300
400
500
600
700

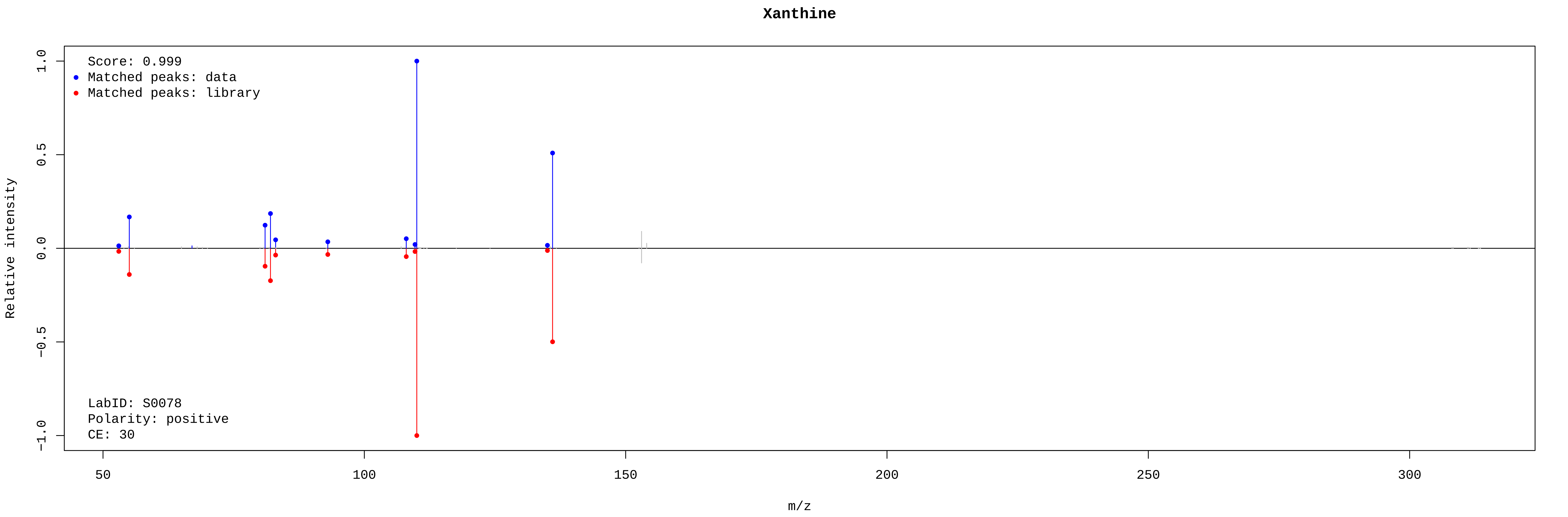

Xanthine
Score: 0.999
Matched peaks: data
Matched peaks: library
LabID: S0078
Polarity: positive
CE: 30
Relative intensity
m/z
1.0
0.5
0.0
-0.5
-1.0
50
100
150
200
250
300

Linoleic acid

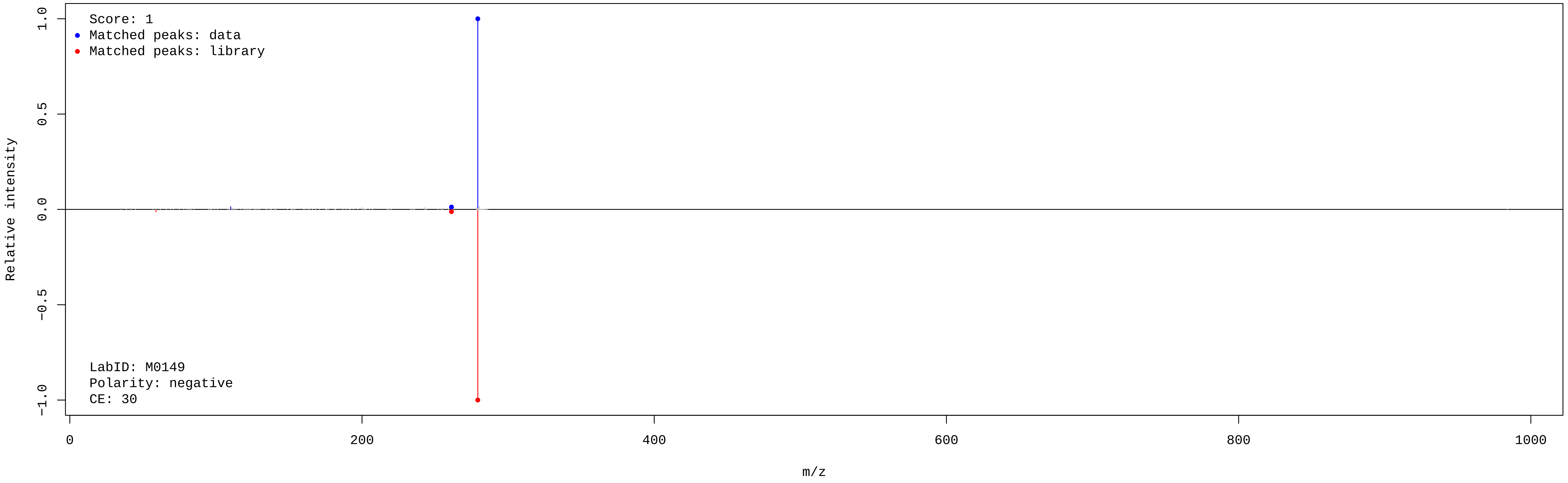

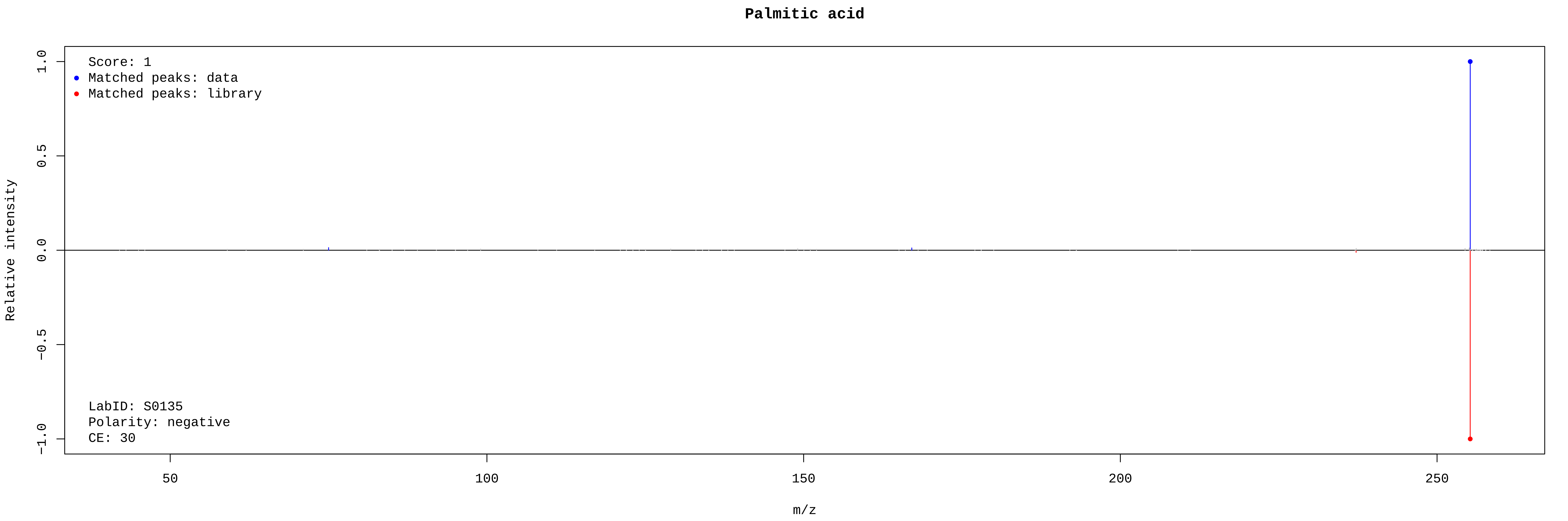
Palmitic acid
Score: 1
Matched peaks: data
Matched peaks: library
LabID: S0135
Polarity: negative
CE: 30
Relative intensity
1.0
0.5
0.0
-0.5
-1.0
50
100
150
200
250
m/z
